# Using remotely monitored patient activity patterns after hospital discharge to predict 30 day hospital readmission: a randomized trial

**DOI:** 10.1038/s41598-023-35201-9

**Published:** 2023-05-22

**Authors:** Mitesh S. Patel, Kevin G. Volpp, Dylan S. Small, Genevieve P. Kanter, Sae-Hwan Park, Chalanda N. Evans, Daniel Polsky

**Affiliations:** 1 Ascension, St. Louis, MO USA; 2 Perelman School of Medicine, University of Pennsylvania, Philadelphia, PA USA; 3 Wharton School, University of Pennsylvania, Philadelphia, PA USA; 4 Crescenz Veterans Affairs Medical Center, Philadelphia, PA USA; 5 Sol Price School of Public Polocy, University of Southern California, Los Angeles, CA USA; 6 Johns Hopkins University, Baltimore, MD USA

**Keywords:** Health policy, Health services, Public health

## Abstract

Hospital readmission prediction models often perform poorly, but most only use information collected until the time of hospital discharge. In this clinical trial, we randomly assigned 500 patients discharged from hospital to home to use either a smartphone or wearable device to collect and transmit remote patient monitoring (RPM) data on activity patterns after hospital discharge. Analyses were conducted at the patient-day level using discrete-time survival analysis. Each arm was split into training and testing folds. The training set used fivefold cross-validation and then final model results are from predictions on the test set. A standard model comprised data collected up to the time of discharge including demographics, comorbidities, hospital length of stay, and vitals prior to discharge. An enhanced model consisted of the standard model plus RPM data. Traditional parametric regression models (logit and lasso) were compared to nonparametric machine learning approaches (random forest, gradient boosting, and ensemble). The main outcome was hospital readmission or death within 30 days of discharge. Prediction of 30-day hospital readmission significantly improved when including remotely-monitored patient data on activity patterns after hospital discharge and using nonparametric machine learning approaches. Wearables slightly outperformed smartphones but both had good prediction of 30-day hospital-readmission.

## Introduction

Nearly 1 in 5 patients discharged from the hospital are readmitted within 30 days^1,2^. Despite significant efforts, models to predict which patients will be readmitted often perform poorly^1,3,4^. A major limitation to these approaches is that they rely heavily on data available up until the time of hospital discharge and do not consider information on patient behaviors at home after they have left the hospital. In addition, most prior models have used traditional regression techniques. Recent systematic reviews suggest that machine learning approaches could provide improved prediction^5,6^.

The use of remote patient monitoring (RPM) devices such as smartphones and wearables is increasing, and prior work has demonstrated they are accurate for tracking daily activity patterns^7,8^. Several pilot studies have demonstrated associations between activity levels and patient outcomes including hospital readmission^9–13^, but evidence from more rigorous and larger clinical trials is lacking. While smartphones are more commonly used by adults, wearables may capture additional measures that smartphones do not, such as sleep patterns^8^. It is unknown whether this additional data collected by wearables will lead to improved prediction compared to data collected by smartphones.

The objective of our study was to evaluate whether hospital readmission prediction models could be improved by incorporating RPM data on activity patterns after hospital discharge and by using machine learning approaches. To evaluate whether there were differences in prediction based on the type of RPM device, we randomly assigned patients discharged from medicine services at two hospitals in Philadelphia to use either their smartphone or a wearable device to track daily activity patterns. Many patients already have a smartphone and these devices have been demonstrated to lead to longer-term utilization for RPM than wearables^14^. However, wearables provide information on behaviors that smartphones do not, such as sleep patterns. Therefore, a head-to-head comparison of smartphones vs. wearables could help to inform future implementation efforts. We also compared modeling techniques using parametric and nonparametric approaches. Nonparametric models are not based on a pre-existing mathematical model or function and instead learn from the data itself^15^, which may give them more flexibility in finding the best prediction model.

## Results

**Figure 1:**
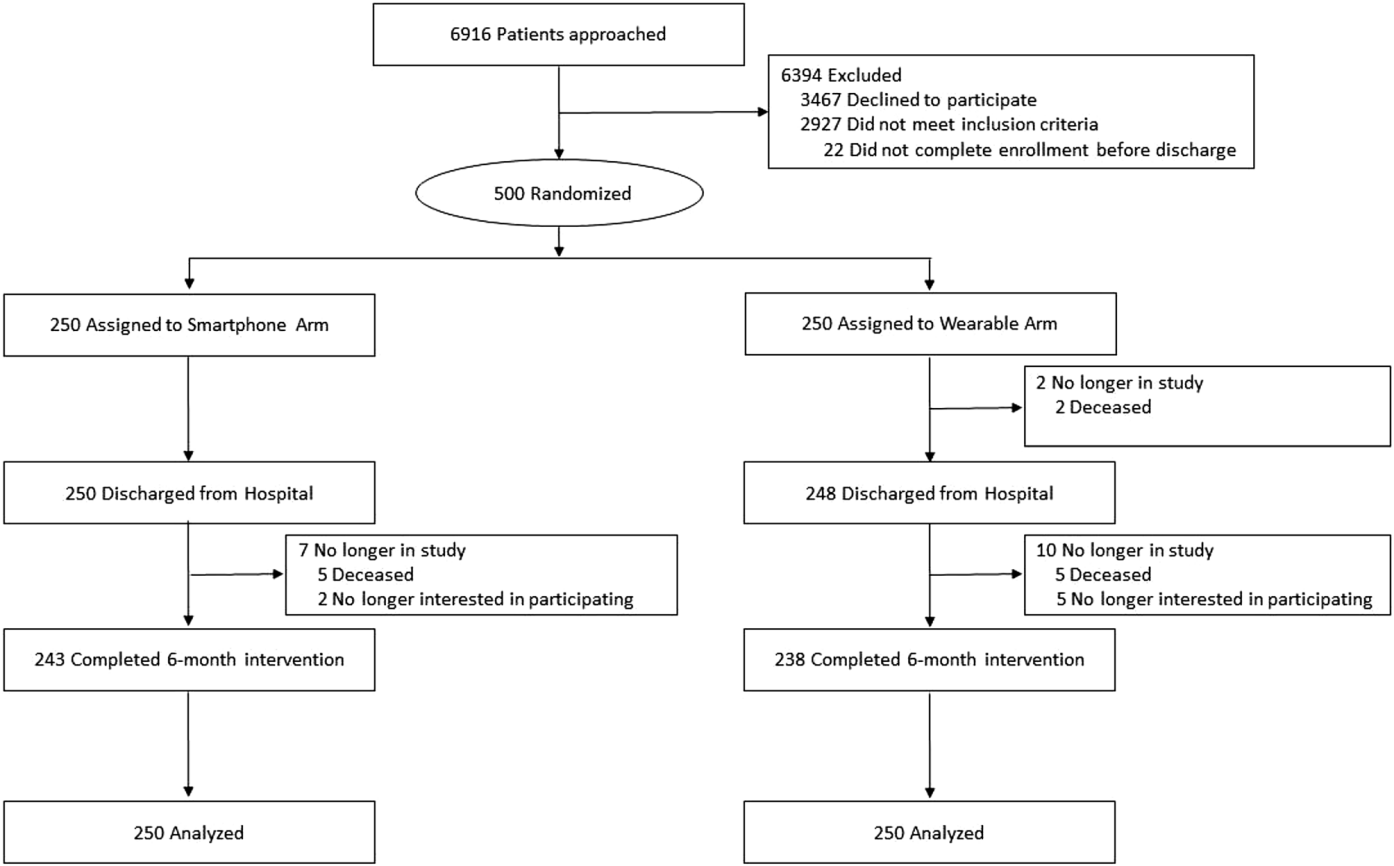
CONSORT diagram. Hospitalized patients were randomly assigned to use either a smartphone application alone or with a wearable device to track activity patterns for 6 months post-discharge.

In this trial, 500 patients were randomized (Fig. 1). Patients had a mean (SD) age of 46.6 (13.7) years and body mass index of 30.9 (9.1); 64.0% (320/500) were female, 43.8% (219/500) were white, 47.0% (235/500) were Black, 28.2% (141/500) were enrolled in Medicare, and 25.6% (128/250) were enrolled in Medicaid. Characteristics were similar between the two study arms (Table 1).

**Table 1:** Patient characteristics.

Characteristic	Smartphone (n = 250)	Wearable (n = 250)
Age, years		
Mean (SD)	46.2 (13.6)	46.9 (13.9)
18–34	61 (24.4)	56 (22.4)
35–49	71 (28.4)	75 (30.0)
50–64	101 (40.4)	94 (37.6)
65 or above	17 (6.8)	25 (10.0)
Female gender	155 (62.0)	165 (66.0)
Race/ethnicity		
White, Non-Hispanic	102 (40.8)	117 (46.8)
Black, Non-Hispanic	125 (50.0)	110 (44.0)
Hispanic	17 (6.8)	11 (4.4)
Other	6 (2.4)	12 (4.8)
Insurance		
Commercial	118 (47.2)	113 (45.2)
Medicare	71 (28.4)	70 (28.0)
Medicaid	61 (24.4)	67 (26.8)
Annual household income, $		
< 30,000	47 (18.8)	52 (20.8)
30,000–59,999	84 (33.6)	77 (30.8)
60,000–99,999	76 (30.4)	70 (28.0)
≥ 100,000	43 (17.2)	51 (20.4)
Marital status		
Single	117 (46.8)	106 (42.4)
Married	92 (36.8)	99 (39.6)
Other	41 (16.4)	45 (18.0)
Education		
Less than high school graduate	14 (5.6)	25 (10.0)
High school graduate	150 (60.0)	146 (58.4)
College graduate or higher	86 (34.4)	79 (31.6)
Body mass index, mean (SD)	30.5 (8.6)	31.3 (9.6)
Charlson Comorbidity Index, median (IQR)	2 (1, 5)	2 (1, 4)

*SD* standard deviation, *IQR* interquartile range.

In the smartphone arm, 18.4% (46/250) were readmitted and 0.4% (1/250) died within 30 days of discharge. In the wearable arm, 13.2% (33/250) were readmitted and 0.8% (2/250) died within 30 days. In the 30 days after discharge, the proportion of patient-days that RPM data was transmitted was greater for physical activity data among the smartphone arm (78.6% for smartphone arm vs 70.8% for wearable arm; *P* for difference < 0.001), but greater for sleep data among the wearable arm (0.3% for smartphone arm vs 39.5% for wearable arm; *P* for difference < 0.001).

**Figure 2:**
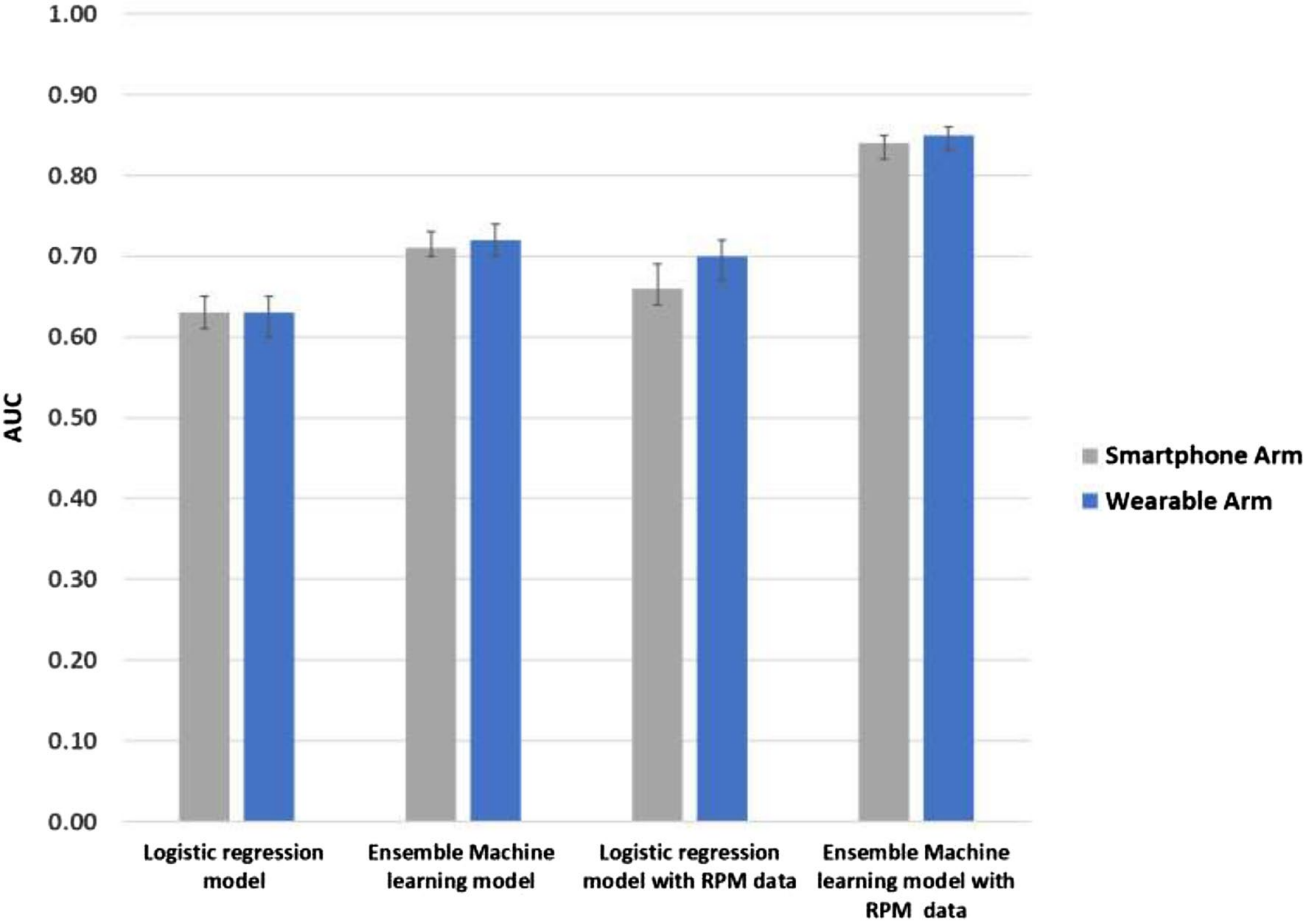
Hospital readmission prediction by regression model, RPM data use, and device arm. Depicted is the area under the receiver operating characteristic curve (AUC) with 95% confidence intervals. Data presented are from the hold-out test set. The machine learning model depicted uses the ensemble machine learning model.

Figure 2 depicts hospital readmission prediction measured using area under the receiver operator characteristic curve (AUC) and 95% confidence intervals by regression model (logistic vs. ensemble machine learning), RPM data (included vs. not included), and device arm (smartphone vs. wearable). Overall, prediction was greater among ensemble models than logistic models and further improved when including RPM data. Results for all five regression models are depicted in Table 2 and described in the following sections.

**Table 2:** Adjusted prediction models for 30-day hospital readmission.

Regression^1^	Standard model^2^			Enhanced model with RPM^3^			Enhanced vs. standard	
	Smartphone AUC (95% CI)	Wearable AUC (95% CI)	P value	Smartphone AUC (95% CI)	Wearable AUC (95% CI)	P value	Smartphone (P value)	Wearable (P value)
Logit	0.63 (0.61, 0.65)	0.63 (0.60, 0.65)	0.07	0.66 (0.64, 0.69)	0.70 (0.67, 0.72)	0.03	< 0.001	< 0.001
Lasso	0.66 (0.64, 0.69)	0.66 (0.63, 0.69)	0.38	0.74 (0.72 0.76)	0.75 (0.73, 0.77)	0.25	< 0.001	< 0.001
Random forest	0.67 (0.65, 0.68)	0.71 (0.68, 0.73)	0.10	0.81 (0.80, 0.83)	0.80 (0.78, 0.82)	0.02	< 0.001	< 0.001
Gradient boosting	0.73 (0.71, 0.75)	0.70 (0.68, 0.72)	0.02	0.80 (0.78, 0.81)	0.84 (0.81, 0.85)	0.6	< 0.001	< 0.001
Ensemble machine learning	0.71 (0.70, 0.73)	0.72 (0.70, 0.74)	0.50	0.84 (0.82 0.85)	0.85 (0.83, 0.86)	0.02	< 0.001	< 0.001

*AUC* area under the receiver operating characteristic curve, *RPM* remote patient monitoring.

^1^Data presented are from predictions on the hold-out test set.

^2^Standard model includes data available until the time of hospital discharge including demographic information, time, hospital length of stay, vitals prior to discharge, and the Charlson Comorbidity Index.

^3^Enhanced model is the standard model plus RPM data on physical activity.

### Standard model with traditional regression

These models used electronic health record data until the time of hospital discharge. As expected, since these models did not include RPM data, prediction was similar between device arms for logistic regression (AUC in smartphone arm, 0.63, 95% CI 0.61 to 0.65; AUC in wearable arm, 0.63, 95% CI 0.60 to 0.65; *P* for difference = 0.07) and lasso regression (AUC in smartphone arm, 0.66, 95% CI 0.64 to 0.69; AUC in wearable arm, 0.66, 95% CI 0.63 to 0.69; *P* = 0.38) (Table 2).

### Standard model with machine learning regression

Prediction increased when using nonparametric machine learning approaches than when using traditional parametric regression techniques (Table 2). In the ensemble machine learning approach, prediction was similar between device arms (AUC in smartphone arm, 0.71, 95% CI 0.70 to 0.73; AUC in wearable arm, 0.72, 95% CI 0.70 to 0.74; *P* = 0.50).

### Enhanced prediction models with RPM data

For all 5 regression approaches, prediction increased significantly when RPM data on physical activity and sleep patterns was included in the models (*P* < 0.001 for each comparison of enhanced vs. standard model) (Table 2). For example, in the standard model using ensemble machine learning approach, the AUC was 0.71 and 0.72 for the smartphone and wearable arm, respectively. When adding RPM data, these AUCs increased to 0.84 and 0.85, respectively.

**Figure 3:**
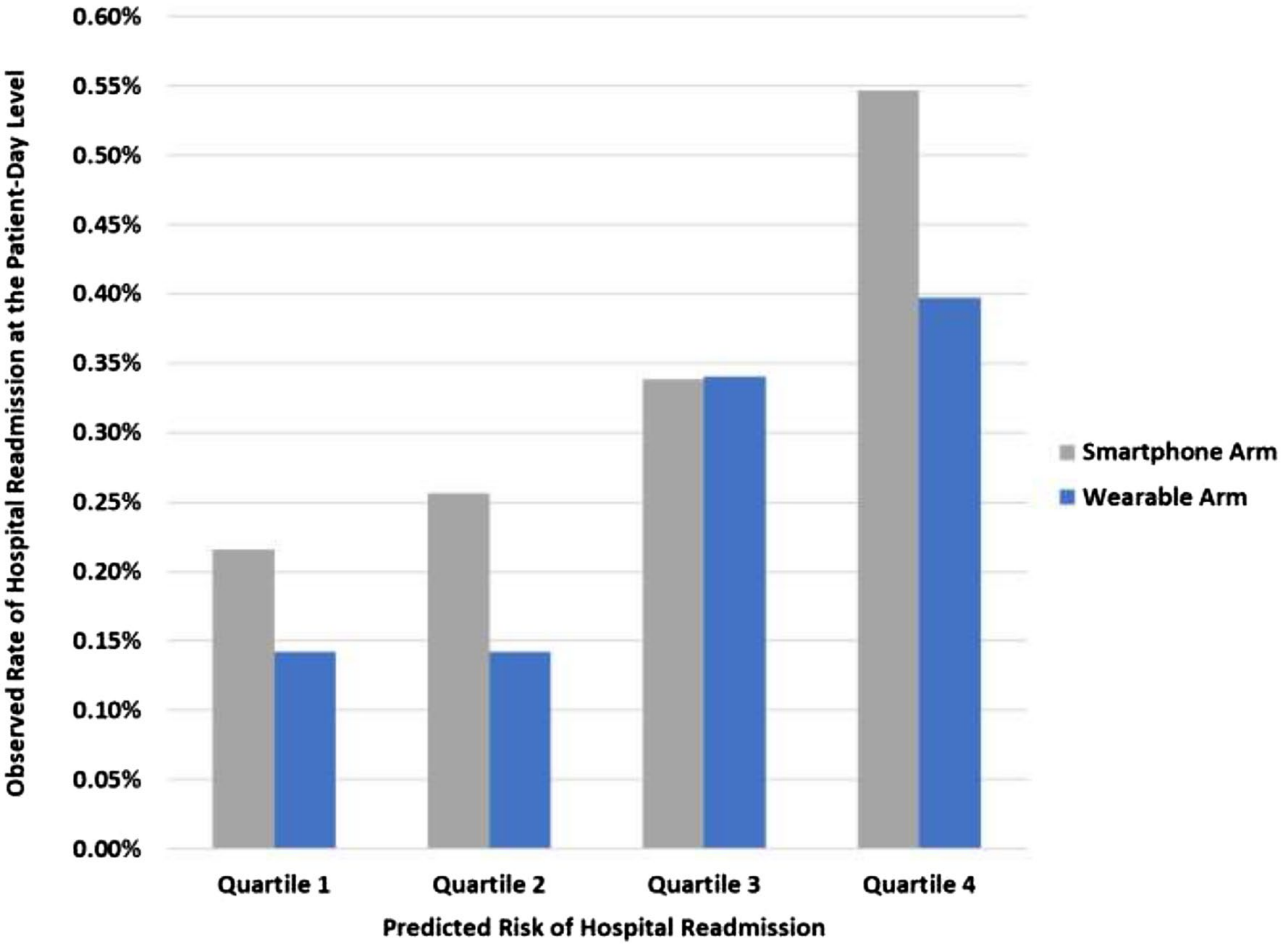
Observed readmission rate by predicted risk quartile for the enhanced model with RPM data and ensemble machine learning. Depicted is the observed rate of hospital readmission at the patient-day level by predicted risk quartile (1 is lowest risk; 4 is highest risk) and by study arm. Data presented use the enhanced model that includes remotely-monitored data and the ensemble machine learning model.

Figure 3 depicts the observed hospital readmission rate at the patient-day level by study arm and quartile of predicted risk from the enhanced model with RPM data using ensemble machine learning. The observed readmission rate increased as predicted risk increased.

### Wearable versus smartphone

In the enhanced models that incorporated RPM data, 4 of the 5 models found that the wearable arm had significantly better prediction than the smartphone arm (Table 2). However, the magnitude of differences between arms were small. For example, in the ensemble machine learning approach, the AUC was 0.84 (95% CI 0.82 to 0.85) in the smartphone arm and 0.85 (95% CI 0.83 to 0.86) in the wearable arm (*P* for difference = 0.02). There were no reported adverse events in the trial.

## Discussion

In this clinical trial, we found that hospital readmission prediction models that used traditional parametric regression approaches with electronic health record data until the time of hospital discharge performed similar to previously published models with AUCs ranging from 0.60 to 0.70^1,3,4^. Prediction improved when including remote patient monitoring (RPM) data on physical activity and sleep patterns after hospital discharge (AUC range: 0.66 to 0.75). The best prediction included RPM data with nonparametric machine learning approaches (AUC range 0.80 to 0.85). In the randomized trial, we found that RPM data from wearables outperformed RPM data from smartphones, but that differences between the two arms were small. To our knowledge, this is one of the first studies of its kind and demonstrates that hospital readmission prediction models could be significantly improved if they used nonparametric machine learning approaches and incorporated RPM data.

Our findings reveal several important insights for future implementation and research efforts. Prediction in the wearable arm was significantly greater than the smartphone arm, but the difference was small (AUC 0.85 for wearable vs. 0.84 for smartphone). Prior work has found that physical activity data collected by these devices is similar in accuracy^7^, but that smartphone device users transmitted RPM data at higher rates than wearable users over time^14^. Indeed, in this study we found those using a smartphone tracked their physical activity patterns at higher rates than those using a wearable (78.6% vs. 70.8%). This might be because patients may stop using a wearable device but do not stop carrying their smartphone. However, smartphone users still need to open the app to transmit physical activity data and smartphones do not track sleep data unless a patient manually reports it. These competing factors of better data quality with sleep data from wearables but less data availability may have led to similar prediction rates between the device arms. In the future, it will be important to compare prediction over a longer-term period such as 90 or 180 days. Since more than 80% of adults have a smartphone^16^, these devices may be a more scalable approach than purchasing wearables for patients to use. Future work should also evaluate wearables that can track additional biometrics such as heart rate and oxygen saturation.

A recent systematic review indicated that machine learning methods showed promise for improving hospital readmission models but that more rigorous evaluation was needed^5^. Another systematic review found that machine learning models of EHR data did slightly outperform traditional parametric approaches with AUCs averaging 0.74 in machine learning models^6^. In our trial, nonparametric machine learning approaches consistently outperformed traditional parametric regression techniques such as logit or lasso models. This was true for both the standard models with data until the time of discharge and enhanced models that incorporated RPM data. While machine learning approaches will need to be further tested in other settings, our findings suggest they could be an important component of efforts to improve prediction of important health outcomes.

This study has several strengths. First, we evaluated an important clinical outcome and used several data sources including electronic health records and state databases to identify hospital readmissions. Second, we enrolled a diverse patient sample that of which 47% were 50 years or older, 53% were Black or Hispanic, and 26% had Medicaid. Third, we evaluated 5 different modeling techniques including traditional parametric and nonparametric machine learning approaches and found consistent results across these models. Fourth, we collected remote patient monitoring data on patient behavior after hospital discharge, which has not traditionally been integrated into hospital readmission prediction models. Fifth, we conducted a pragmatic, randomized trial to provide a rigorous assessment of the difference in prediction between two of the most common devices used for remote patient monitoring.

### Limitations

This study also has limitations. First, the sample included patients with a mean age of about 46 years from medicine services at one health system who were being discharged to home and less than 10% of those approached agreed to participate, which limits generalizability. Future studies should evaluate a broader sample of patients. Second, the analyses evaluated hospital readmissions within 30 days. Future studies should evaluate prediction of rehospitalization at longer periods of time after discharge. Third, data on hospital readmissions was obtained from Penn Medicine and a database on hospitalizations in the State of Pennsylvania. We did not have access to readmissions that occurred outside of Pennsylvania. Fourth, while participant characteristics between study arms were similar, the readmission rate was lower in the wearable arm. This imbalance may have been due to random chance and the small sample size. However, it does not impact the main findings that RPM data with machine learning improved prediction. Fifth, RPM data was limited to measures of physical activity and sleep. We did not have data on heart rate or other biometrics which can be captured by some wearable devices. Nonetheless, we did find improvements in prediction with these RPM measures and future studies could evaluate if additional biometric data can further improvement.

## Conclusions

Prediction of 30-day hospital readmission significantly improved when including remotely-monitored patient data on activity patterns after hospital discharge and using nonparametric machine learning approaches. Wearables slightly outperformed smartphones but both had good prediction. Since many patients use smartphones and wearables, data from remote patient monitoring devices could be incorporated more broadly into prediction models to identify the patients at highest risk of hospital readmission.

## Methods

### Study design

PREDICT (Prediction using a Randomized Evaluation of Data collection Integrated through Connected Technologies) was a 2-arm randomized clinical trial conducted remotely after patients were discharged from inpatient medicine services at two Penn Medicine hospitals in Philadelphia to their home (ClinicalTrials.gov number, NCT02983812). The trial was conducted was conducted from January 23, 2017 to December 7, 2019. Patients were randomly assigned to use either a smartphone application alone or with a wearable device to collect data on activity patterns for 6 months. Trends in device utilization during this time period have been previously published^14^. In this study, we present the pre-specified primary analysis which evaluated whether remote patient monitoring (RPM) data could be used to improve prediction of hospital readmission within 30 days of discharge.

The trial interventions were conducted using Way to Health, a research technology platform at the University of Pennsylvania used previously for remote-monitoring of activity patterns^14,17–21^. The trial protocol was approved by the University of Pennsylvania IRB, has been published^22^, and is available (Supplement 1). This trial and methods were performed in accordance with the appropriate guidelines and regulations including the Consolidated Standards of Reporting Trials (CONSORT) reporting guideline (Supplement 2).

### Participants

Patients admitted to medicine services at two hospitals at Penn Medicine in Philadelphia (Hospital of the University of Pennsylvania and Penn Presbyterian Medical Center) were identified as potential participants using the electronic health record (EPIC) and approached in the hospital by the study team.

Patients were eligible for the trial if they were 18 years or older, had a smartphone compatible with the Withings HealthMate smartphone application, had no current medical condition which prohibited them from ambulating or plan for a medical procedure over the next 6 months that would prohibit them from ambulating, planned to be discharged to home, able to speak and read English, and able to provide informed consent. Patients were excluded if pregnant, already participating in another physical activity study or if they did not reside in the States of Pennsylvania or New Jersey.

Interested patients used a computer from the study team to create an account on the Way to Health technology platform, provide informed consent, and selected whether to receive study communications by text message, email, interactive voice recording, or a combination. Patients then completed series of survey assessments including baseline information and validated questionnaires.

### Data

Patient data obtained from Way to Health included demographics collected by survey during initial hospitalization (age, gender, race/ethnicity, education, marital status, annual household income, and body mass index) and RPM activity patterns transmitted from the smartphone application and wearable including daily measures of step counts, distance, calories burned (active and total), minutes of activity (soft and moderate intensity), minutes of sleep (total, light, and deep), minutes to fall asleep, minutes awake, and number of times awakened. The smartphone application alone did not track sleep but patients in that arm could manually input sleep on their own. Data obtained from the electronic health record included insurance type, the Charlson Comorbidity Index^23^, vitals (measures for the following were obtained as the value available closest to the time of discharge: temperature, heart rate, systolic and diastolic blood pressures, oxygen saturation, respirations per minute), hospital length of stay, hospital discharge date, date of any hospitalizations within 6 months of discharge, and if applicable, death date. Means and standard deviations of RPM and EHR data are available (Supplementary Tables 1, 2). Data on hospitalizations in Pennsylvania that occurred outside of Penn Medicine were obtained from the Pennsylvania Health Care Cost Containment Council.

### Randomization

Patients were randomized electronically using block sizes of two and stratified by one of the following six primary conditions for admission: acute myocardial infarction or coronary heart disease, chronic obstructive pulmonary disease, congestive heart failure, diabetes, pneumonia, or any other condition. These conditions were selected to represent the leading primary diagnoses associated with hospital readmissions in Pennsylvania for which we hypothesized activity patterns could inform readmission rates. All investigators, statisticians, and data analysts were blinded to arm assignments until the study and analysis were completed.

### Interventions

Patients assigned to the smartphone arm were set up in the hospital with the Withings Health Mate smartphone application which used accelerometers in the smartphone to track physical activity patterns. They were asked to open the application at least once a day to sync the device. These patients were told they would receive a wearable device after their 6-month period completed. Patients assigned to the wearable device arm were also setup in the hospital with the Withings Steel which had a battery that lasted up to 8 months without recharging. In addition to tracking physical activity patterns, the wearable also tracked sleep patterns. Patients were asked to wear the device as much as possible including while sleeping and to sync it with the Withings Health Mate Smartphone application at least once a day.

In both arms throughout the 6-month period, patients were sent a reminder to sync their device if data had not been transmitted for four consecutive days. All patients received $50 to enroll and $50 to complete the trial.

### Outcome measures

The primary outcome was prediction of readmission to the hospital or death within 30 days of discharge.

### Statistical analysis

All randomly assigned patients were included in the intention-to-treat analysis. For each patient and on each day of the study (patient-day level), we obtained RPM activity data. Data could be missing for any day if the patient did not use the device, did not sync it to upload data, or the device did not capture it (e.g. smartphones did not track sleep data). We also excluded outliers (top and bottom 1 percentile) in activity data and coded them as missing. To account for missing values in the RPM data, we included a dichotomous missing-value indicator variable for each RPM data variable^24^. We also conducted multiple imputation by chain equations (MICE) with five sets of imputations that used a mixed effect model with the following predictors: study arm, day of the study, calendar month, weekend or weekday, age, sex, race/ethnicity, insurance type, education, marital status, annual household income, body mass index, hospital length of stay, Charlson comorbidity index, and vitals (temperature, heart rate, systolic and diastolic blood pressures, oxygen saturation, respirations per minute)^25^. Missing data rates are available (Supplementary Table 1). Since the amount of missing data itself could be an indicator of readmission risk, we also developed a variable for the proportion of days of data captured. Because more changes in RPM values may be better predictors than earlier changes, we created lagging indicator variables at the patient-day level that used a weighted average of data over the previous 3 days as follows: 1 day prior (0.9 weight), 2 days prior (0.9^2^ or 0.81 weight), and 3 days prior (0.9^3^ or 0.73 weight). RPM data was censored on the day of readmission or death.

We used a discrete-time survival analysis and evaluated hospital readmission or death within 30 days of discharge at the patient-day level as the unit of observation. The sample was split into training and testing folds for each arm that were mutually exclusive at the patient level in a 3:2 ratio. The training set used fivefold cross-validation and then final model results are from predictions on the test set.

We fit a standard model that comprised data available up to the time of hospital discharge including demographic information (age, gender, race/ethnicity, education, marital status, annual household income, and body mass index), time (calendar month and year), hospital length of stay, vitals near discharge (temperature, heart rate, systolic and diastolic blood pressures, oxygen saturation, respirations per minute), and Charlson Comorbidity Index. We fit an enhanced model with RPM data that included the same variables as the standard model as well as the following: daily measures of step counts, distance, calories burned (active and total), minutes of activity (soft and moderate intensity), minutes of sleep (total, light, and deep), minutes awake, and number of times awakened. The enhanced model with RPM also including missing-value and lagged indicators for each RPM measure, as previously described. For all models, we compared traditional parametric regression (logit and lasso) to nonparametric machine learning approaches (random forest, gradient boosting, and ensemble machine learning). The ensemble machine learning model used a combination of logit, lasso, random forest, and gradient boosting. Hyperparameter specification is described in Supplementary Table 3.

Model performance was assessed using the area under the receiver operator characteristic curve (AUC)^26,27^. The Delong method was used to test for differences in AUC between study arms and models^28^. To estimate 95% confidence intervals we used 1000 bootstrapped samples. We also estimated the observed rate of readmission by quartile of predicted risk. Analyses were conducted using Scikit-learn version 0.24.1 package, XGBoost version 0.9 package, and Lifelines version 0.25.7 package with Python version 3.7.9 (Python Software Foundation). We used 2-sided hypothesis tests (level of significance, P < 0.05).

## Supplementary Information

Supplementary Information 1.

Supplementary Information 2.

Supplementary Information 3.

## Data Availability

The datasets generated and/or analysed during the current study are not publicly available because we do not have IRB approval to share them since they contain patient information. Dr. Patel had full access to all the data in the study and takes responsibility for the integrity of the data and the accuracy of the data analysis.
