# Supplementary material for: Using remotely monitored patient activity patterns after hospital discharge to predict 30 day hospital readmission: a randomized trial
Source: Sci Rep. 2023 May 22;13:8258. doi: 10.1038/s41598-023-35201-9 (PMC10203290; doi:10.1038/s41598-023-35201-9)
Supplement: Supplementary file 1 — Supplementary Information 1. [file 41598_2023_35201_MOESM1_ESM.docx]

This Supplement contains the following items

1. Original protocol, final protocol, summary of changes
2. Original statistical analysis plan, final statistical analysis plan, summary of changes

Original Protocol

**Prediction using a Randomized Evaluation of Data collection Integrated through Connected Technologies**

Short Title:

**The PREDICT Trial**

December 15, 2016

**Outline**

1. Abstract

2. Overall objectives

3. Aims

3.1 Primary outcome

3.2 Secondary outcomes

4. Background

5. Study design

5.1 Design

5.2 Study duration

5.3 Target population

5.4 Accrual

5.5 Key inclusion criteria

5.6 Key exclusion criteria

6. Subject recruitment

7. Subject compensation

8. Study procedures

8.1 Consent

8.2 Procedures

9. Analysis plan

10. Investigators

11. Human research protection

11.1 Data confidentiality

11.2 Subject confidentiality

11.3 Subject privacy

11.4 Data disclosure

11.5 Data safety and monitoring

11.6 Risk/benefit

11.6.1 Potential study risks

11.6.2 Potential study benefits

11.6.3 Risk/benefit assessment

12. Bibliography

**1. Abstract**

Many hospital readmissions could be prevented if higher risk patients were identified and effective interventions then targeted towards these individuals. However, most existing claims-based predictive models perform poorly and do not provide timely and actionable information. In this study, we will prospectively enroll patients for data collection to design prediction models that integrate claims data (inpatient, outpatient, and pharmacy), electronic health record data (on clinical, social, and behavioral indicators), validated surveys (on risk preferences, social support, personality, medication adherence and self-reported physical activity prior to admission) and use wearable devices or smartphones to collect patient-generated data (physical activity and sleep patterns). Patients will be randomized to use either a smartphone or a wearable activity tracking device to capture patient-generated health data.

**2. Overall objectives**

The overall objective of the study is to develop algorithms for the dynamic and timely prediction of health care utilization using a multimodal, integrated dataset from insurer and pharmacy claims, electronic health records, and patient-generated health data. We will test different methods of collecting patient-generated health data including self-reported surveys, smartphones, and a wearable activity tracking device.

**3. Aims**

*3.1 Primary outcome*

The primary outcome measure will be 30-day hospital readmission.

*3.2 Secondary outcome*

The secondary outcome measures include 90-day readmission, 6-month re-hospitalization and health care cost utilization within 6 months after discharge.

**4. Background**

Among patients with chronic conditions, approximately 1 in 5 is readmitted to the hospital within 30 days after discharge. Underserved and minority populations often have even higher rates of readmission. Many of these readmissions could be prevented if higher risk patients were identified and effective interventions then targeted towards these individuals. However, most existing claims-based predictive models perform poorly and do not provide timely and actionable information. There has been a large amount of prior research conducted using inpatient claims data to design prediction models for hospital readmission. However, most algorithms designed to identify these high risk individuals have c- statistics in the range of 0.60-0.70, indicating that such algorithms are better than chance but leave a lot of room for improvement. In addition, disparities in out-of-hospital care and medication adherence may exist and contribute to higher rates of readmission among minority and lower income populations. We will develop prediction algorithms integrate claims data (inpatient, outpatient, and pharmacy), electronic health record data (on clinical, social, and behavioral indicators), validated surveys and use wearable devices or smartphones to collect patient-generated data (physical activity and sleep patterns).

**5. Study design**

*5.1 Design*

We will conduct a clinical trial in which we monitor patient-generated health data from 500 patients discharged from one of the University of Pennsylvania Health System hospitals for 6 months. Patients will be randomly assigned to track their data using a smartphone app (which collects step counts) or a wearable activity tracker (which collects step counts and sleep patterns/duration). There will be no intervention for either group, both are being passively monitored. The group assigned to use the smartphone app will receive a wearable activity tracker at the end of the 6 month study.

The University of Pennsylvania Health System will help us to design a real-time list of patient admitted to the hospitals that fit our inclusion criteria. Research coordinators will visit these patients to describe the study and assess their interest in participating. Research coordinators will help all interested participants to create an account on the Way to Health online research platform to begin the enrollment process.

All patients will complete several validated surveys including the Medical Outcome Study (MOS) Social Support, DOSPERT risk preferences, Morisky Medication Adherence (MMAS-8), the Big Five personality test, and short form of the International Physical Activity Questionnaire (IPAQ).

Patients will complete informed consent to allow us to access their health insurance claims data from Independence Blue Cross (IBC), Pennsylvania Health Care Cost Containment Council (PHC4), New Jersey Department of Health, and CVS, their electronic health record data (inpatient and outpatient) from the University of Pennsylvania Health System, their financial data (credit score and timeliness to pay bills) from the University of Pennsylvania Health System or a credit vender, and patient-generated health data collected by their smartphone or wearable activity tracking device.

*5.2 Study duration*

The study period is 6 months in duration. The randomized controlled trial is expected to take about 12 months to complete enrollment with 6 months of follow-up data collection. Analysis is then expected to take about 6 months to complete after data from third parties are received.

*5.3 Target population*

The study population will be drawn from patients admitted to the Hospital of the University of Pennsylvania or Penn Presbyterian Medical Center.

*5.4 Accrual*

The study population will be drawn from adults admitted to one of the University of Pennsylvania Health System hospitals. Patients will be invited to participate by a member of the research team prior to hospital discharge.

*5.5 Key inclusion criteria*

To be eligible patients must:

1. Be 18 years or older
2. Be able to provide informed consent
3. Be admitted to the Hospital of the University of Pennsylvania or Penn Presbyterian Medical Center
4. Have a smartphone or tablet compatible with the Withings Health Mate smartphone application
5. Have no medical condition which prohibits them from ambulating or plan for any medical procedure over the next 6 months that would prohibit them from ambulating
6. Plan to be discharged to home

*5.6 Key exclusion criteria*

Patients not residing in the State of Pennsylvania or New Jersey.

**6. Subject recruitment**

The University of Pennsylvania Health System will help us to design a real-time list of patient admitted to the hospitals that fit our inclusion criteria. Research coordinators will visit these patients to describe the study and assess their interest in participating. Research coordinators will help all interested participants to create an account on the Way to Health online research platform to begin the enrollment process. The research coordinators will assist with smartphone app or wearable activity tracking device setup accordingly, and participant will begin transmitting patient-generated health data.

**7. Subject compensation**

Participants will be compensated $50 for enrolling in the study and $50 for transmitting patient-generated health data through 6 months.

**8. Study procedures**

*8.1 Consent*

Informed consent will be obtained in writing and through the Way to Health study website. Participants will be allowed time to read through the informed consent and a member of the research team will be available for questions. Participants will be provided with a copy of the combined informed consent/ HIPAA form. They will be able to access and print a copy of the informed consent form from the Way to Health online platform as well. Participants will be instructed that they may reach out to the study team with questions and that, since participation is voluntary, they are able to drop out of the study at any time.

*8.2 Procedures*

Patients admitted to one of the hospitals at the University of Pennsylvania Health System will be invited to participate in the study prior to hospital discharge by a member of the research team. Patients will be asked to complete an initial screening questionnaire to assess eligibility. If eligible, participants will then complete an informed consent and HIPAA authorization form. Once consented, participants will complete a series of validated surveys as described earlier. Participants will then be randomized into being passively monitored by smartphone or wearable activity tracking device. Randomization will be stratified based one the five conditions of interest to ensure balance between study arms. After randomization the coordinator will inform the participant of their assignment and read the instructions for their study group. The coordinator will assist the participant in setting up their device as applicable per group assignment, and the participant will begin transmitting patient-generated health data to the study team. Participants will receive a reminder to sync their data using their smartphone application if they have not done so for 4 days in a row.

**9. Analysis plan**

We will conduct preliminary descriptive analyses to compare univariate associations between levels and changes in levels of patient-generated health data and health care utilization.

A standard model will be developed using inpatient claims data by fitting a multivariate logistic regression model to each of the binary dependent outcome variables using hospital and time fixed effects (month and year), and including independent variables for patient demographics, comorbidities, and length of stay in the hospital.

An enhanced model will be developed using the design of the standard model but also incorporating independent variables that represent data on medication adherence, from the electronic medical record on clinical, social, and behavioral factors, from validated surveys, and data from activity tracking devices.

We will perform tests between the enhanced (using data from smartphones and wearables) and standard models to identify significant predicators of the outcome measures that will inform the final model. We will also compare the cross-validated c-statistic between the standard and enhanced models using the replication method. To validate the final model, we will randomly split the participants in several cohorts (e.g. 5 samples of 100 participants) and using all one cohort to validate and the others to train the model. We will perform this several times until each cohort has been used to validate the model. We will evaluate the c-statistic using the replication method.

We will compare predictors of the outcomes and the cross-validated c-statistics between the final model for participants using smartphones compare to participants using wearable devices using the replication method.

All hypothesis tests will be 2-sided and use a significance level of P < 0.05.

The study analysis and prediction models will be informed by another retrospective study that has already been approved by the University of Pennsylvania Institutional Review Board (Protocol # 824908).

**10. Investigators**

Mitesh Patel, MD, MBA, MS is the Principal Investigator (PI) and is an Assistant Professor of Medicine and Health Care Management at the Perelman School of Medicine and The Wharton School at the University of Pennsylvania. He has past experience leading clinical trials in the inpatient and outpatient setting, specifically with technology-based interventions. He currently spends 80% of his effort on research and 20% on clinical and teaching activities.

**11. Human research protection**

*11.1 Data confidentiality*

Computer-based files will only be made available to personnel involved in the study through the use of access privileges and passwords. Wherever feasible, patient identifiers will be removed from study-related information. Precautions are in place to ensure the data are secure by using passwords and encryption.

*11.2 Subject confidentiality*

Computer-based files will only be made available to personnel involved in the study through the use of access privileges and passwords. Wherever feasible, patient identifiers will be removed from study- related information. Precautions are in place to ensure the data are secure by using passwords and encryption. Data use agreements are in progress with Independence Blue Cross, Pennsylvania Health Care Cost Containment Council (PHC4), New Jersey Department of Health, and CVS Health, as noted above in Data Management section. Research material that is obtained will be used for research purposes only. All study staff will be reminded to appreciate the confidential nature of the data collected and contained in these databases. Way to Health (WTH) is hosted on site at The University of Pennsylvania (UPenn) and is protected by a secure firewall. Once a participant is in this system, they will be given a unique study identification number (ID). Any datasets and computer files that leave the firewall will be stripped of all identifiers and individuals will be referred to by their study ID. The study ID will also be used on all analytical files. Please see attached document (WTH database security text) for full database security details.

The Penn Medicine Academic Computing Services (PMACS) will be the hub for the hardware and database infrastructure that will support the project. The PMACS is a joint effort of the University of Pennsylvania's Abramson Cancer Center, the Cardiovascular Institute, the Department of Pathology, and the Leonard Davis Institute. The PMACS provides a secure computing environment for a large volume of highly sensitive data, including clinical, genetic, socioeconomic, and financial information. Among the IT projects currently managed by PMACS are: (1) the capture and organization of complex, longitudinal clinical data via web and clinical applications portals from cancer patients enrolled in clinical trials; (2) the integration of genetic array databases and clinical data obtained from patients with cardiovascular disease; (3) computational biology and cytometry database management and analyses; (4) economic and health policy research using Medicare claims from over 40 million Medicare beneficiaries. PMACS requires all users of data or applications on PMACS servers to complete a PMACS-hosted cybersecurity awareness course annually, which stresses federal data security policies under data use agreements with the university. The curriculum includes Health Insurance Portability and Accountability Act (HIPAA) training and covers secure data transfer, passwords, computer security habits and knowledge of what constitutes misuse or inappropriate use of the server. We will implement multiple, redundant protective measures to guarantee the privacy and security of the participant data. All investigators and research staff with direct access to the identifiable data will be required to undergo annual responsible conduct of research, cybersecurity, and HIPAA certification in accordance with University of Pennsylvania regulations. All data for this project will be stored on the secure/firewalled servers of the PMACS Data Center, in data files that will be protected by multiple password layers. These data servers are maintained in a guarded facility behind several locked doors, with very limited physical access rights. They are also cyber-protected by extensive firewalls and multiple layers of communication encryption. Electronic access rights are carefully controlled by University of Pennsylvania system managers.

*11.3 Subject privacy*

As in the retrospective data analysis in protocol 824908, we will use subject name, address and date of birth to integrate data into a single database linked at the individual level. In cases where further information is needed to link data, we will use other identifiers such as SSN. SSN will only be used in this case by case basis where linking is not possible. Requests for use of SSN for this purpose will be submitted as a separate modification. All of these data will be stored in an encrypted database that conforms to applicable data security standards. Once linking is complete and identifiers are no longer needed, we will replace them with a de-identified unique patient number so that we can identify unique patients without using identifiers.

For the randomized controlled trial described here, computer-based files will only be made available to personnel involved in the study through the use of access privileges and passwords. A secure pin number will be used to de-identify data transmitted from the smartphone to the Way to Health platform. Precautions are in place to ensure the data are secure by using passwords and encryption. Individual identifiers (such as name, address, and SSN for compensation) will be stored in a single password protected system that is accessible only to study research, analysis and IT staff. This system, Way to Health (WTH), is hosted on site at The University of Pennsylvania (UPenn) and is protected by a secure firewall. Once a participant is in this system, they will be given a unique study identification number (ID). Any datasets and computer files that leave the firewall will be stripped of all identifiers and individuals will be referred to by their study ID. The study ID will also be used on all analytical files. Please see attached document (WTH database security text) for full database security details.

*11.4 Data disclosure*

Participant SSNs will only be shared with the US government if a W-9 form is submitted for tax purposes and will never be disclosed to any other partnering organizations. Participant information may be disclosed to the following companies for the purposes specified:

• Wells Fargo Bank (to coordinate study payments)

• Twilio Cloud Communications (to send study messages to participants)

• Qualtrics (to collect subject answers to survey questions)

• Federal and state agencies (for example, the Department of Health and Human Services, the National Institutes of Health, and/or the Office for Human Research Protections), or other domestic or foreign government bodies if required by law and/or necessary for oversight purposes.

•Withings (to record activity from the smartphone app or wearable device)

•Credit Reporting Agency (to collect credit worthiness. This will not negatively affect the subject's credit score)

•Independence Blue Cross (to access health insurance claims and pharmacy benefit claims for research purposes only)

•CVS/Caremark (to access pharmacy benefit claims for research purposes only)

The privacy policies of these companies are available here:

• Wells Fargo: https://www.wellsfargo.com/privacy_security/privacy/individuals

• Twilio Cloud Communications: http://www.twilio.com/legal/privacy

• Qualtrics: http://www.qualtrics.com/privacy-statement/

• Withings: <https://www2.withings.com/ca/en/legal/privacy>

• Independence Blue Cross: https://www.ibx.com/privacy/index.html

• CVS/Caremark: privacy policy at https://www.caremark.com/wps/portal/

*11.5 Data safety and monitoring*

The Principal Investigator and Research Coordinators will closely monitor the safety, privacy, and data integrity of the study. Because the study consists of passive observation and no clinical intervention, there will be no additional data safety monitoring. Patients will be provided contact information for study staff and if adverse events are identified, events will be reported and brought to the PI’s attention.

*11.6 Risk/benefit*

*11.6.1 Potential study risks*

All data described previously will be protected as described in the Subject confidentiality section. There is minimal risk to subjects as there is minimal risk of breach of data. Our team has extensive experience working with these types of data.

*11.6.2 Potential study benefits*

This unique collaboration and application of big data analysis could improve the health of Pennsylvanians by predicting clinical events earlier and more accurately than ever before. The study may have greatest impact in addressing health disparities because prediction moves beyond the walls of the hospital and into homes and communities, where the most vulnerable patients face the greatest challenges. Through harnessing Big Data for more precise and timely prediction of actionable events, this application fills a critical gap in Pennsylvania's health care system in its efforts to improve quality and ameliorate health disparities.

*11.6.3 Risk/benefit assessment*

There is minimal risk of breach of data and appropriate measures have been taken. Therefore, we believe the risk/benefit assessment is favorable given the potential insights that could be yielded from the findings of this study.

Bibliography

1.Pennsylvania Health Care Cost Containment Council. Hopsital Performance Report, 2013 Data, Southeastern, PA., 2013. (Accessed at http://www.phc4.org/reports/hpr/13/.)

3.Joynt KE, Jha AK. Characteristics of hospitals receiving penalties under the Hospital Readmissions Reduction Program. JAMA : the journal of the American Medical Association 2013;309:342-3.

4.Joynt KE, Orav EJ, Jha AK. Thirty-day readmission rates for Medicare beneficiaries by race and site of care. JAMA : the journal of the American Medical Association 2011;305:675-81.

5.Jack BW, Chetty VK, Anthony D, et al. A reengineered hospital discharge program to decrease rehospitalization. Annals of Internal Medicine 2009;150:178-87.

6.Coleman EA, Parry C, Chalmers S, Min SJ. The care transitions intervention: results of a randomized controlled trial. Arch Intern Med 2006;166:1822-8.

7.Naylor MD, Brooten D, Campbell R, et al. Comprehensive discharge planning and home follow-up of hospitalized elders: a randomized clinical trial. JAMA : the journal of the American Medical Association 1999;281:613-20.

8.Kansagara D, Englander H, Salanitro A, et al. Risk prediction models for hospital readmission: a systematic review. JAMA : the journal of the American Medical Association 2011;306:1688-98.

9.Keyhani S, Myers LJ, Cheng E, Hebert P, Williams LS, Bravata DM. Effect of clinical and social risk factors on hospital profiling for stroke readmission: a cohort study. Ann Intern Med 2014;161:775-84.

125.Hosmer DW, Lemeshow S. applied Logistic Regression. 2nd ed. New York, NY: John Wiley & Sons; 2000.

130.Thomas AJ, Eberly LE, Davey Smith G, Neaton JD. ZIP-code-based versus tract-based income measures as long-term risk-adjusted mortality predictors. American journal of epidemiology 2006;164:586-90.

133.Tabak YP, Johannes RS, Silber JH. Using automated clinical data for risk adjustment: development and validation of six disease-specific mortality predictive models for pay-for-performance. Med Care 2007;45:789-805.

134.Fiks AG, Alessandrini EA, Luberti AA, Ostapenko S, Zhang X, Silber JH. Identifying factors predicting immunization delay for children followed in an urban primary care network using an electronic health record. Pediatrics 2006;118:e1680-6.

135.Green AR, Carney DR, Pallin DJ, et al. Implicit bias among physicians and its prediction of thrombolysis decisions for black and white patients. J Gen Intern Med 2007;22:1231-8.

136.Iezzoni LI. Risk Adjustment for Measuring Healthcare Outcomes. 4th ed. Chicago, IL: Health Administration Press; 2012.

137.Elixhauser A, Steiner C, Harris DR, Coffey RM. Comorbidity measures for use with administrative data. Med Care 1998;36:8-27.

138.Donze J, Aujesky D, Williams D, Schnipper JL. Potentially avoidable 30-day hospital readmissions in medical patients: derivation and validation of a prediction model. JAMA internal medicine 2013;173:632-8.

139.Krumholz HM, Chen YT, Wang Y, Vaccarino V, Radford MJ, Horwitz RI. Predictors of readmission among elderly survivors of admission with heart failure. American heart journal 2000;139:72-7.

Final Protocol

**Prediction using a Randomized Evaluation of Data collection Integrated through Connected Technologies**

Short Title:

**The PREDICT Trial**

May 16, 2017

**Outline**

1. Abstract

2. Overall objectives

3. Aims

3.1 Primary outcome

3.2 Secondary outcomes

4. Background

5. Study design

5.1 Design

5.2 Study duration

5.3 Target population

5.4 Accrual

5.5 Key inclusion criteria

5.6 Key exclusion criteria

6. Subject recruitment

7. Subject compensation

8. Study procedures

8.1 Consent

8.2 Procedures

9. Analysis plan

10. Investigators

11. Human research protection

11.1 Data confidentiality

11.2 Subject confidentiality

11.3 Subject privacy

11.4 Data disclosure

11.5 Data safety and monitoring

11.6 Risk/benefit

11.6.1 Potential study risks

11.6.2 Potential study benefits

11.6.3 Risk/benefit assessment

12. Bibliography

**1. Abstract**

Many hospital readmissions could be prevented if higher risk patients were identified and effective interventions then targeted towards these individuals. However, most existing claims-based predictive models perform poorly and do not provide timely and actionable information. In this study, we will prospectively enroll patients for data collection to design prediction models that integrate claims data (inpatient, outpatient, and pharmacy), electronic health record data (on clinical, social, and behavioral indicators), validated surveys (on risk preferences, social support, personality, medication adherence and self-reported physical activity prior to admission) and use wearable devices or smartphones to collect patient-generated data (physical activity and sleep patterns). Patients will be randomized to use either a smartphone or a wearable activity tracking device to capture patient-generated health data.

**2. Overall objectives**

The overall objective of the study is to develop algorithms for the dynamic and timely prediction of health care utilization using a multimodal, integrated dataset from insurer and pharmacy claims, electronic health records, and patient-generated health data. We will test different methods of collecting patient-generated health data including self-reported surveys, smartphones, and a wearable activity tracking device.

**3. Aims**

*3.1 Primary outcome*

The primary outcome measure will be 30-day hospital readmission.

*3.2 Secondary outcome*

The secondary outcome measures include 90-day readmission, 6-month re-hospitalization and health care cost utilization within 6 months after discharge.

**4. Background**

Among patients with chronic conditions, approximately 1 in 5 is readmitted to the hospital within 30 days after discharge. Underserved and minority populations often have even higher rates of readmission. Many of these readmissions could be prevented if higher risk patients were identified and effective interventions then targeted towards these individuals. However, most existing claims-based predictive models perform poorly and do not provide timely and actionable information. There has been a large amount of prior research conducted using inpatient claims data to design prediction models for hospital readmission. However, most algorithms designed to identify these high risk individuals have c- statistics in the range of 0.60-0.70, indicating that such algorithms are better than chance but leave a lot of room for improvement. In addition, disparities in out-of-hospital care and medication adherence may exist and contribute to higher rates of readmission among minority and lower income populations. We will develop prediction algorithms integrate claims data (inpatient, outpatient, and pharmacy), electronic health record data (on clinical, social, and behavioral indicators), validated surveys and use wearable devices or smartphones to collect patient-generated data (physical activity and sleep patterns).

**5. Study design**

*5.1 Design*

We will conduct a clinical trial in which we monitor patient-generated health data from 500 patients discharged from one of the University of Pennsylvania Health System hospitals for 6 months. Patients will be randomly assigned to track their data using a smartphone app (which collects step counts) or a wearable activity tracker (which collects step counts and sleep patterns/duration). There will be no intervention for either group, both are being passively monitored. The group assigned to use the smartphone app will receive a wearable activity tracker at the end of the 6 month study.

The University of Pennsylvania Health System will help us to design a real-time list of patient admitted to the hospitals that fit our inclusion criteria. Research coordinators will visit these patients to describe the study and assess their interest in participating. Research coordinators will help all interested participants to create an account on the Way to Health online research platform to begin the enrollment process.

All patients will complete several validated surveys including the Medical Outcome Study (MOS) Social Support, DOSPERT risk preferences, Morisky Medication Adherence (MMAS-8), the Big Five personality test, and short form of the International Physical Activity Questionnaire (IPAQ).

Patients will complete informed consent to allow us to access their health insurance claims data from Independence Blue Cross (IBC), Pennsylvania Health Care Cost Containment Council (PHC4), New Jersey Department of Health, and CVS, their electronic health record data (inpatient and outpatient) from the University of Pennsylvania Health System, their financial data (credit score and timeliness to pay bills) from the University of Pennsylvania Health System or a credit vender, and patient-generated health data collected by their smartphone or wearable activity tracking device.

*5.2 Study duration*

The study period is 6 months in duration. The randomized controlled trial is expected to take about 12 months to complete enrollment with 6 months of follow-up data collection. Analysis is then expected to take about 6 months to complete after data from third parties are received.

*5.3 Target population*

The study population will be drawn from patients admitted to the Hospital of the University of Pennsylvania or Penn Presbyterian Medical Center.

*5.4 Accrual*

The study population will be drawn from adults admitted to one of the University of Pennsylvania Health System hospitals. Patients will be invited to participate by a member of the research team prior to hospital discharge.

*5.5 Key inclusion criteria*

To be eligible patients must:

1. Be 18 years or older
2. Be able to provide informed consent
3. Be admitted to the Hospital of the University of Pennsylvania or Penn Presbyterian Medical Center
4. Have a smartphone or tablet compatible with the Withings Health Mate smartphone application
5. Have no medical condition which prohibits them from ambulating or plan for any medical procedure over the next 6 months that would prohibit them from ambulating
6. Plan to be discharged to home

*5.6 Key exclusion criteria*

Patients not residing in the State of Pennsylvania or New Jersey.

**6. Subject recruitment**

The University of Pennsylvania Health System will help us to design a real-time list of patient admitted to the hospitals that fit our inclusion criteria. Research coordinators will visit these patients to describe the study and assess their interest in participating. Research coordinators will help all interested participants to create an account on the Way to Health online research platform to begin the enrollment process. The research coordinators will assist with smartphone app or wearable activity tracking device setup accordingly, and participant will begin transmitting patient-generated health data.

**7. Subject compensation**

Participants will be compensated $50 for enrolling in the study and $50 for transmitting patient-generated health data through 6 months.

**8. Study procedures**

*8.1 Consent*

Informed consent will be obtained in writing and through the Way to Health study website. Participants will be allowed time to read through the informed consent and a member of the research team will be available for questions. Participants will be provided with a copy of the combined informed consent/ HIPAA form. They will be able to access and print a copy of the informed consent form from the Way to Health online platform as well. Participants will be instructed that they may reach out to the study team with questions and that, since participation is voluntary, they are able to drop out of the study at any time.

*8.2 Procedures*

Patients admitted to one of the hospitals at the University of Pennsylvania Health System will be invited to participate in the study prior to hospital discharge by a member of the research team. Patients will be asked to complete an initial screening questionnaire to assess eligibility. If eligible, participants will then complete an informed consent and HIPAA authorization form. Once consented, participants will complete a series of validated surveys as described earlier. Participants will then be randomized into being passively monitored by smartphone or wearable activity tracking device. Randomization will be stratified based one the five conditions of interest to ensure balance between study arms. After randomization the coordinator will inform the participant of their assignment and read the instructions for their study group. The coordinator will assist the participant in setting up their device as applicable per group assignment, and the participant will begin transmitting patient-generated health data to the study team. Participants will receive a reminder to sync their data using their smartphone application if they have not done so for 4 days in a row.

**9. Analysis plan**

We will conduct preliminary descriptive analyses to compare univariate associations between levels and changes in levels of patient-generated health data and health care utilization.

A standard model will be developed using inpatient claims data by fitting a multivariate logistic regression model to each of the binary dependent outcome variables using hospital and time fixed effects (month and year), and including independent variables for patient demographics, comorbidities, and length of stay in the hospital.

An enhanced model will be developed using the design of the standard model but also incorporating independent variables that represent data on medication adherence, from the electronic medical record on clinical, social, and behavioral factors, from validated surveys, and data from activity tracking devices.

We will perform tests between the enhanced (using data from smartphones and wearables) and standard models to identify significant predicators of the outcome measures that will inform the final model. We will also compare the cross-validated c-statistic between the standard and enhanced models using the replication method. To validate the final model, we will randomly split the participants in several cohorts (e.g. 5 samples of 100 participants) and using all one cohort to validate and the others to train the model. We will perform this several times until each cohort has been used to validate the model. We will evaluate the c-statistic using the replication method.

We will compare predictors of the outcomes and the cross-validated c-statistics between the final model for participants using smartphones compare to participants using wearable devices using the replication method.

All hypothesis tests will be 2-sided and use a significance level of P < 0.05.

The study analysis and prediction models will be informed by another retrospective study that has already been approved by the University of Pennsylvania Institutional Review Board (Protocol # 824908).

**10. Investigators**

Mitesh Patel, MD, MBA, MS is the Principal Investigator (PI) and is an Assistant Professor of Medicine and Health Care Management at the Perelman School of Medicine and The Wharton School at the University of Pennsylvania. He has past experience leading clinical trials in the inpatient and outpatient setting, specifically with technology-based interventions. He currently spends 80% of his effort on research and 20% on clinical and teaching activities.

**11. Human research protection**

*11.1 Data confidentiality*

Computer-based files will only be made available to personnel involved in the study through the use of access privileges and passwords. Wherever feasible, patient identifiers will be removed from study-related information. Precautions are in place to ensure the data are secure by using passwords and encryption.

*11.2 Subject confidentiality*

Computer-based files will only be made available to personnel involved in the study through the use of access privileges and passwords. Wherever feasible, patient identifiers will be removed from study- related information. Precautions are in place to ensure the data are secure by using passwords and encryption. Data use agreements are in progress with Independence Blue Cross, Pennsylvania Health Care Cost Containment Council (PHC4), New Jersey Department of Health, and CVS Health, as noted above in Data Management section. Research material that is obtained will be used for research purposes only. All study staff will be reminded to appreciate the confidential nature of the data collected and contained in these databases. Way to Health (WTH) is hosted on site at The University of Pennsylvania (UPenn) and is protected by a secure firewall. Once a participant is in this system, they will be given a unique study identification number (ID). Any datasets and computer files that leave the firewall will be stripped of all identifiers and individuals will be referred to by their study ID. The study ID will also be used on all analytical files. Please see attached document (WTH database security text) for full database security details.

The Penn Medicine Academic Computing Services (PMACS) will be the hub for the hardware and database infrastructure that will support the project. The PMACS is a joint effort of the University of Pennsylvania's Abramson Cancer Center, the Cardiovascular Institute, the Department of Pathology, and the Leonard Davis Institute. The PMACS provides a secure computing environment for a large volume of highly sensitive data, including clinical, genetic, socioeconomic, and financial information. Among the IT projects currently managed by PMACS are: (1) the capture and organization of complex, longitudinal clinical data via web and clinical applications portals from cancer patients enrolled in clinical trials; (2) the integration of genetic array databases and clinical data obtained from patients with cardiovascular disease; (3) computational biology and cytometry database management and analyses; (4) economic and health policy research using Medicare claims from over 40 million Medicare beneficiaries. PMACS requires all users of data or applications on PMACS servers to complete a PMACS-hosted cybersecurity awareness course annually, which stresses federal data security policies under data use agreements with the university. The curriculum includes Health Insurance Portability and Accountability Act (HIPAA) training and covers secure data transfer, passwords, computer security habits and knowledge of what constitutes misuse or inappropriate use of the server. We will implement multiple, redundant protective measures to guarantee the privacy and security of the participant data. All investigators and research staff with direct access to the identifiable data will be required to undergo annual responsible conduct of research, cybersecurity, and HIPAA certification in accordance with University of Pennsylvania regulations. All data for this project will be stored on the secure/firewalled servers of the PMACS Data Center, in data files that will be protected by multiple password layers. These data servers are maintained in a guarded facility behind several locked doors, with very limited physical access rights. They are also cyber-protected by extensive firewalls and multiple layers of communication encryption. Electronic access rights are carefully controlled by University of Pennsylvania system managers.

*11.3 Subject privacy*

As in the retrospective data analysis in protocol 824908, we will use subject name, address and date of birth to integrate data into a single database linked at the individual level. In cases where further information is needed to link data, we will use other identifiers such as SSN. SSN will only be used in this case by case basis where linking is not possible. Requests for use of SSN for this purpose will be submitted as a separate modification. All of these data will be stored in an encrypted database that conforms to applicable data security standards. Once linking is complete and identifiers are no longer needed, we will replace them with a de-identified unique patient number so that we can identify unique patients without using identifiers.

For the randomized controlled trial described here, computer-based files will only be made available to personnel involved in the study through the use of access privileges and passwords. A secure pin number will be used to de-identify data transmitted from the smartphone to the Way to Health platform. Precautions are in place to ensure the data are secure by using passwords and encryption. Individual identifiers (such as name, address, and SSN for compensation) will be stored in a single password protected system that is accessible only to study research, analysis and IT staff. This system, Way to Health (WTH), is hosted on site at The University of Pennsylvania (UPenn) and is protected by a secure firewall. Once a participant is in this system, they will be given a unique study identification number (ID). Any datasets and computer files that leave the firewall will be stripped of all identifiers and individuals will be referred to by their study ID. The study ID will also be used on all analytical files. Please see attached document (WTH database security text) for full database security details.

*11.4 Data disclosure*

Participant SSNs will only be shared with the US government if a W-9 form is submitted for tax purposes and will never be disclosed to any other partnering organizations. Participant information may be disclosed to the following companies for the purposes specified:

• Wells Fargo Bank (to coordinate study payments)

• Twilio Cloud Communications (to send study messages to participants)

• Qualtrics (to collect subject answers to survey questions)

• Federal and state agencies (for example, the Department of Health and Human Services, the National Institutes of Health, and/or the Office for Human Research Protections), or other domestic or foreign government bodies if required by law and/or necessary for oversight purposes.

•Withings (to record activity from the smartphone app or wearable device)

•Credit Reporting Agency (to collect credit worthiness. This will not negatively affect the subject's credit score)

•Independence Blue Cross (to access health insurance claims and pharmacy benefit claims for research purposes only)

•CVS/Caremark (to access pharmacy benefit claims for research purposes only)

The privacy policies of these companies are available here:

• Wells Fargo: https://www.wellsfargo.com/privacy_security/privacy/individuals

• Twilio Cloud Communications: http://www.twilio.com/legal/privacy

• Qualtrics: http://www.qualtrics.com/privacy-statement/

• Withings: <https://www2.withings.com/ca/en/legal/privacy>

• Independence Blue Cross: https://www.ibx.com/privacy/index.html

• CVS/Caremark: privacy policy at https://www.caremark.com/wps/portal/

*11.5 Data safety and monitoring*

The Principal Investigator and Research Coordinators will closely monitor the safety, privacy, and data integrity of the study. Because the study consists of passive observation and no clinical intervention, there will be no additional data safety monitoring. Patients will be provided contact information for study staff and if adverse events are identified, events will be reported and brought to the PI’s attention.

*11.6 Risk/benefit*

*11.6.1 Potential study risks*

All data described previously will be protected as described in the Subject confidentiality section. There is minimal risk to subjects as there is minimal risk of breach of data. Our team has extensive experience working with these types of data.

*11.6.2 Potential study benefits*

This unique collaboration and application of big data analysis could improve the health of Pennsylvanians by predicting clinical events earlier and more accurately than ever before. The study may have greatest impact in addressing health disparities because prediction moves beyond the walls of the hospital and into homes and communities, where the most vulnerable patients face the greatest challenges. Through harnessing Big Data for more precise and timely prediction of actionable events, this application fills a critical gap in Pennsylvania's health care system in its efforts to improve quality and ameliorate health disparities.

*11.6.3 Risk/benefit assessment*

There is minimal risk of breach of data and appropriate measures have been taken. Therefore, we believe the risk/benefit assessment is favorable given the potential insights that could be yielded from the findings of this study.

Bibliography

1.Pennsylvania Health Care Cost Containment Council. Hopsital Performance Report, 2013 Data, Southeastern, PA., 2013. (Accessed at http://www.phc4.org/reports/hpr/13/.)

3.Joynt KE, Jha AK. Characteristics of hospitals receiving penalties under the Hospital Readmissions Reduction Program. JAMA : the journal of the American Medical Association 2013;309:342-3.

4.Joynt KE, Orav EJ, Jha AK. Thirty-day readmission rates for Medicare beneficiaries by race and site of care. JAMA : the journal of the American Medical Association 2011;305:675-81.

5.Jack BW, Chetty VK, Anthony D, et al. A reengineered hospital discharge program to decrease rehospitalization. Annals of Internal Medicine 2009;150:178-87.

6.Coleman EA, Parry C, Chalmers S, Min SJ. The care transitions intervention: results of a randomized controlled trial. Arch Intern Med 2006;166:1822-8.

7.Naylor MD, Brooten D, Campbell R, et al. Comprehensive discharge planning and home follow-up of hospitalized elders: a randomized clinical trial. JAMA : the journal of the American Medical Association 1999;281:613-20.

8.Kansagara D, Englander H, Salanitro A, et al. Risk prediction models for hospital readmission: a systematic review. JAMA : the journal of the American Medical Association 2011;306:1688-98.

9.Keyhani S, Myers LJ, Cheng E, Hebert P, Williams LS, Bravata DM. Effect of clinical and social risk factors on hospital profiling for stroke readmission: a cohort study. Ann Intern Med 2014;161:775-84.

125.Hosmer DW, Lemeshow S. applied Logistic Regression. 2nd ed. New York, NY: John Wiley & Sons; 2000.

130.Thomas AJ, Eberly LE, Davey Smith G, Neaton JD. ZIP-code-based versus tract-based income measures as long-term risk-adjusted mortality predictors. American journal of epidemiology 2006;164:586-90.

133.Tabak YP, Johannes RS, Silber JH. Using automated clinical data for risk adjustment: development and validation of six disease-specific mortality predictive models for pay-for-performance. Med Care 2007;45:789-805.

134.Fiks AG, Alessandrini EA, Luberti AA, Ostapenko S, Zhang X, Silber JH. Identifying factors predicting immunization delay for children followed in an urban primary care network using an electronic health record. Pediatrics 2006;118:e1680-6.

135.Green AR, Carney DR, Pallin DJ, et al. Implicit bias among physicians and its prediction of thrombolysis decisions for black and white patients. J Gen Intern Med 2007;22:1231-8.

136.Iezzoni LI. Risk Adjustment for Measuring Healthcare Outcomes. 4th ed. Chicago, IL: Health Administration Press; 2012.

137.Elixhauser A, Steiner C, Harris DR, Coffey RM. Comorbidity measures for use with administrative data. Med Care 1998;36:8-27.

138.Donze J, Aujesky D, Williams D, Schnipper JL. Potentially avoidable 30-day hospital readmissions in medical patients: derivation and validation of a prediction model. JAMA internal medicine 2013;173:632-8.

139.Krumholz HM, Chen YT, Wang Y, Vaccarino V, Radford MJ, Horwitz RI. Predictors of readmission among elderly survivors of admission with heart failure. American heart journal 2000;139:72-7.

Summary of Protocol Changes

May 16, 2017. Two changes were made on May 16, 2017 to increase enrollment rates. First, we expanded the criteria to include all medical diagnoses rather than the original set of five. Second, we offered those in control a wearable device after the 6 months follow-up period ended. This allowed us to tell all potential participants that they would get a wearable, either now or in 6 months.

Original Statistical Analysis Plan

We will conduct preliminary descriptive analyses to compare univariate associations between levels and changes in levels of patient-generated health data and health care utilization.

A standard model will be developed using inpatient claims data by fitting a multivariate logistic regression model to each of the binary dependent outcome variables using hospital and time fixed effects (month and year), and including independent variables for patient demographics, comorbidities, and length of stay in the hospital.

An enhanced model will be developed using the design of the standard model but also incorporating independent variables that represent data on medication adherence, from the electronic medical record on clinical, social, and behavioral factors, from validated surveys, and data from activity tracking devices.

We will perform tests between the enhanced (using data from smartphones and wearables) and standard models to identify significant predicators of the outcome measures that will inform the final model. We will also compare the cross-validated c-statistic between the standard and enhanced models using the replication method. To validate the final model, we will randomly split the participants in several cohorts (e.g. 5 samples of 100 participants) and using all one cohort to validate and the others to train the model. We will perform this several times until each cohort has been used to validate the model. We will evaluate the c-statistic using the replication method.

We will compare predictors of the outcomes and the cross-validated c-statistics between the final model for participants using smartphones compare to participants using wearable devices using the replication method.

All hypothesis tests will be 2-sided and use a significance level of P < 0.05.

The study analysis and prediction models will be informed by another retrospective study that has already been approved by the University of Pennsylvania Institutional Review Board (Protocol # 824908).

Final Statistical Analysis Plan

We will conduct preliminary descriptive analyses to compare univariate associations between levels and changes in levels of patient-generated health data and health care utilization.

A standard model will be developed using inpatient claims data by fitting a multivariate logistic regression model to each of the binary dependent outcome variables using hospital and time fixed effects (month and year), and including independent variables for patient demographics, comorbidities, and length of stay in the hospital.

An enhanced model will be developed using the design of the standard model but also incorporating independent variables that represent data on medication adherence, from the electronic medical record on clinical, social, and behavioral factors, from validated surveys, and data from activity tracking devices.

We will perform tests between the enhanced (using data from smartphones and wearables) and standard models to identify significant predicators of the outcome measures that will inform the final model. We will also compare the cross-validated c-statistic between the standard and enhanced models using the replication method. To validate the final model, we will randomly split the participants in several cohorts (e.g. 5 samples of 100 participants) and using all one cohort to validate and the others to train the model. We will perform this several times until each cohort has been used to validate the model. We will evaluate the c-statistic using the replication method.

We will compare predictors of the outcomes and the cross-validated c-statistics between the final model for participants using smartphones compare to participants using wearable devices using the replication method.

All hypothesis tests will be 2-sided and use a significance level of P < 0.05.

The study analysis and prediction models will be informed by another retrospective study that has already been approved by the University of Pennsylvania Institutional Review Board (Protocol # 824908).

Statistical Analysis Plan Summary of Changes

None
