# Supplementary material for: Using remotely monitored patient activity patterns after hospital discharge to predict 30 day hospital readmission: a randomized trial
Source: Sci Rep. 2023 May 22;13:8258. doi: 10.1038/s41598-023-35201-9 (PMC10203290; doi:10.1038/s41598-023-35201-9)
Supplement: Supplementary file 3 — Supplementary Information 3. [file 41598_2023_35201_MOESM3_ESM.docx]

**Supplement 3**

**Supplementary Appendix**

Table of Contents

**Supplementary Table 1**. Mean values, standard deviations, and missing data rates for remote patient monitoring variables

**Supplementary Table 2**. Mean values, standard deviations, and missing data rates for electronic health record variables

**Supplementary Table 3**. Hyperparameter specification for prediction models

**Supplementary Table 1. Mean values, standard deviations, and missing data rates for remote patient monitoring variables**


*Data are at the patient-day level and are censored on the day of hospital readmission or death
**Abbreviations: SD=standard deviation

**Supplementary Table 2. Mean values, standard deviations, and missing data rates for electronic health record variables**

*Data are at the patient level

**Abbreviations: SD=standard deviation

**Supplementary Table 3**. Hyperparameter specification for prediction models

## Hyperparameters for Ensemble Machine Learning (Meta Learner)

We used the stacked generalization method for combining individual machine learning algorithms (also known as individual learners) to reduce their biases. More precisely, the predictions of each individual learner were stacked together and used as input to a final classifier algorithm to compute the prediction. The final estimator was trained through cross-validation and the following settings.

| Hyperparameter | Parameter Value |
| --- | --- |
| Cross Validation Splitting Strategy | 5-fold cross validation without refitting individual leaners |
| Methods called for each individual learner | ‘predict_proba’ |
| Classifier used to combine individual learners | ‘LogisticRegression’ |

## Hyperparameter Tuning for Individual Learners

For the three ensemble tree algorithms (gradient boosting, random forest, XG boosting), we used an exhaustive grid search (i.e., all grid points tested) and 5-fold cross validation on the training cohort to determine hyperparameters. Note that each different modeling combination – each model (standard and enhanced) and each trial arm – hyperparameters separately tuned. The area under the receiver operating characteristic curve (AUROC) was used as a maximization objective function for all the hyperparameter tuning processes of this study. For anything else that were not specified in our hyperparameter space table, we let each algorithm take its default parameters as are.

### Hyperparameter Space for Individual Learners

|  | Random Forest | Gradient Boosting | XG Boosting |
| --- | --- | --- | --- |
| No. of trees | {100, 500, 1000, 2000} | Same as left | Same as left |
| Algorithm to determine maximum no. of features to consider at every split | { ‘sqrt’, ‘log2’} | Same as left | {’hist’,’exact’,’approx’} |
| Max no. of levels in tree | {2, 3, 4, unlimited} | Same as left | N/A |
| Min no. of samples required to split a node | {2, 5, 10, 15, 20} | Same as left | N/A |
| Min No. of samples required at each leaf node | {1, 2, 5, 10, 15} | Same as left | N/A |
| min sum of weights of all obs. required in a child | N/A | N/A | {0, 1, 5, 10} |
| MIN loss reduction requried to make a split | N/A | N/A | {0, 1, 5, 10} |
| L1 regularization term on weights | N/A | N/A | {0, 20, 50, 100} |
| L2 Regularization term on weights | N/A | N/A | {0, 0.1, 0.2, 0.5, 1.0} |
| The fraction of samples to be used for fitting the individual tree | N/A | {60%, 80%, 100%} | Same as left |
| Learning rate | N/A | {0.55%, 1%, 2%} | Same as left |

### Optimized Hyperparameters

| Standard Models | RF (Smartphone Arm) | RF (Wearable arm) | GB (smartphone arm) | GB (wearable arm) | XGB (Smartphone arm) | XGB (WEARABLE ARM) |
| --- | --- | --- | --- | --- | --- | --- |
| No. of trees | 1000 | 1000 | 2000 | 500 | 1000 | 500 |
| Algorithm to determine maximum no. of features to consider at every split | Log2 | Log2 | Log2 | Log2 | Exact | Approx |
| Max no. of levels in tree | Unlimited | Unlimited | Unlimited | Unlimited | N/A | N/A |
| Min no. of samples required to split a node | 2 | 5 | 20 | 2 | N/A | N/A |
| Min No. of samples required at each leaf node | 1 | 1 | 5 | 1 | N/A | N/A |
| min sum of weights of all obs. required in a child | N/A | N/A | N/A | N/A | 0 | 0 |
| MIN loss reduction requried to make a split | N/A | N/A | N/A | N/A | 1 | 1 |
| L1 regularization term on weights | N/A | N/A | N/A | N/A | 20 | 20 |
| L2 Regularization term on weights | N/A | N/A | N/A | N/A | 0.1 | 0.1 |
| The fraction of samples to be used for fitting the individual tree | N/A | N/A | 80% | 60% | 80% | 60% |
| Learning rate | N/A | N/A | 0.5% | 2% | 2% | 2% |

| enhanced models | RF (Arm X) | RF (ARM Y) | GB (Arm X) | GB (ARM Y) | XGB (Smartphone arm) | XGB (WEARABLE ARM) |
| --- | --- | --- | --- | --- | --- | --- |
| No. of trees | 500 | 100 | 2000 | 1000 | 1000 | 1000 |
| Algorithm to determine maximum no. of features to consider at every split | Log2 | Log2 | Log2 | Log2 | Exact | Hist |
| Max no. of levels in tree | Unlimited | Unlimited | Unlimited | 4 | N/A | N/A |
| Min no. of samples required to split a node | 2 | 2 | 15 | 5 | N/A | N/A |
| Min No. of samples required at each leaf node | 1 | 1 | 5 | 1 | N/A | N/A |
| min sum of weights of all obs. required in a child | N/A | N/A | N/A | N/A | 0 | 1 |
| MIN loss reduction requried to make a split | N/A | N/A | N/A | N/A | 1 | 5 |
| L1 regularization term on weights | N/A | N/A | N/A | N/A | 20 | 50 |
| L2 Regularization term on weights | N/A | N/A | N/A | N/A | 0.2 | 0.5 |
| The fraction of samples to be used for fitting the individual tree | N/A | N/A | 80% | 80% | 60% | 60% |
| Learning rate | N/A | N/A | 1% | 1% | 2% | 1% |
